# Barriers to healthcare predict reduced health-related quality of life in autistic adults without intellectual disability

**DOI:** 10.1177/13623613241275406

**Published:** 2024-09-11

**Authors:** Nicole David, Pascal Rahlff, Hannah König, Sophia Dückert, Petia Gewohn, Frank Erik, Kai Vogeley, Daniel Schöttle, Alexander Konnopka, Holger Schulz, Judith Peth

**Affiliations:** 1 University Medical Center Hamburg-Eppendorf, Germany; 2 University of Cologne, Germany

**Keywords:** adults, autism spectrum disorders, health services, quality of life

## Abstract

Autistic adults frequently show increased physical or mental health conditions and impairments in social, occupational, and other life domains. Accordingly, they often report reduced health-related quality of life. Yet, numerous barriers prevent effective healthcare for autistic adults. To date, the impact of healthcare barriers on health-related quality of life in autism has not been investigated. We, thus, sought to investigate the health-related quality of life of autistic adults without intellectual disability in Germany and analyze previously reported and novel predictors of health-related quality of life including healthcare barriers using multiple linear regression. Within a nationwide online survey, 311 autistic adults completed the Short-Form Health Survey and the Barriers to Healthcare Checklist. Demographic, clinical, and healthcare-related predictors were also assessed. Participants showed below-average mental and significantly reduced physical health-related quality of life compared to a German normative sample. Several independent variables differentially predicted mental and physical health-related quality of life. Healthcare barriers resulted as the only predictor, which explained variations in both domains: the more barriers autistic adults experienced, the lower their mental and physical health-related quality of life. Barriers emerged as the strongest predictor for mental health-related quality of life. Our results raise awareness for the barriers autistic adults experience in getting appropriate healthcare and the possible consequences for the individual. Improved healthcare access might contribute to better health-related quality of life in autism.

**Lay abstract:**

Health-related quality of life reflects a person’s perspective on their well-being in physical, mental, social, work-related, and other aspects of health or life. Autistic adults typically report difficulties in many or all of these domains and, thus, often experience their health-related quality of life being reduced. Nonetheless, they do not obtain the professional support they need and report barriers to accessing or receiving appropriate healthcare. We know little about the impact of barriers to healthcare on health-related quality of life in autistic adults. In the present study, 311 autistic adults without intellectual disability in Germany completed an online survey on their current health-related quality of life and the number of barriers to healthcare they experience. In addition, they were asked about their personal and clinical background as well as about the amount of healthcare and support they recently received. We investigated how this information and, particularly, barriers to healthcare explained variations in individual levels of health-related quality of life. We found that barriers to healthcare, compared to most other variables, were a strong predictor of health-related quality of life: The more barriers autistic adults reported, the lower their experienced psychological and physical well-being. To our knowledge, this is the first paper to examine the relationship between barriers to healthcare and health-related quality of life in autism. Our results suggest that healthcare providers need to become aware of the barriers individuals with autism have in seeking and getting healthcare. Improved access to services might contribute to better health-related quality of life in autistic adults.

## Introduction

Health-related quality of life (HRQOL) is a multi-faceted construct that refers to the subjectively perceived well-being in physical, mental, social, work-related, and other domains of life (de Wit & Hajos, 2013). Considering the patient’s perspective and including these dimensions in the notion of health represented a paradigm shift in healthcare, particularly medicine (Bullinger & Quitmann, 2014), and was based on the World Health Organization’s definition of health as “a state of complete physical, mental, and social well-being and not merely the absence of disease.” Meanwhile, HRQOL has become increasingly important as a so-called “patient-reported outcome” for various health conditions (Megari, 2013). As such, current guidelines also highlighted HRQOL as an important outcome in autism spectrum disorder (ASD) (National Institute for Health and Care Excellence [NICE], 2012), as autistic adults are often affected in social and work-related domains of life (Espeloer et al., 2023; Howlin, 2013). Regardless of considerable heterogeneity in the spectrum—with some autistic adults living independently, being married, and socially integrated—there is a ubiquitous need for psychosocial support (David et al., 2022; Lord et al., 2022). Compromised health, as indicated by increased rates of somatic and mental illness, has been reported (Croen et al., 2015; Kohane et al., 2012; Vohra et al., 2017; Williams & Gotham, 2022). Thus, HRQOL represents a relevant outcome in autistic adults, which—as a patient-reported outcome that captures the first-person perspective and strengthens the patient’s involvement—also addresses the increased need of autistic adults for participation during treatment (Duckert et al., 2023).

Although research in autistic adults—as opposed to children—has only recently increased, there already is a body of evidence on HRQOL in autistic adults (Ayres et al., 2018; Kim & Bottema-Beutel, 2019; Mason et al., 2018; Mason, Mackintosh, et al., 2019; van Heijst & Geurts, 2015). Overall, most international evidence suggests poorer HRQOL in autistic adults (cf. Oakley et al., 2021). Current evidence on HRQOL in autistic adults in Germany is limited to one study, which also reported reduced HRQOL in a small sample of adolescents or young autistic adults (Kamp-Becker et al., 2010). Studies on HRQOL in autistic adults often differ in terms of sample size but also in the outcome measure (Ayres et al., 2018; Evers et al., 2022). The World Health Organization Quality of Life-BREF (WHOQOL-Group, 1998) and Short-Form Health Survey (SF-36/12/8; Ware & Sherbourne, 1992) are among the most widely used generic HRQOL measures. Studies using these measures in autistic adults showed particularly lower outcomes in the domains of psychological health/ mental HRQOL, physical health/ HRQOL, and social relationships (e.g., Jennes-Coussens et al., 2006; Kamio et al., 2013; Kamp-Becker et al., 2010; Mason et al., 2018; van Heijst & Geurts, 2015). Existing meta-analyses, however, strongly suggest that HRQOL can be considered generally reduced in autistic adults regardless of such methodological differences (van Heijst & Geurts, 2015).

In an attempt to understand this reduced HRQOL in autistic adults, potential predictors have been investigated. Demographic and autism-related variables are among the most investigated predictors of HRQOL in autistic adults. For example, (female) gender has often been associated with reduced HRQOL (Braden et al., 2022; Kamio et al., 2013; Khanna et al., 2014; Knüppel et al., 2018; Mason et al., 2018), while age did not result as significant predictor of HRQOL in recent meta-regression analyses (Kim & Bottema-Beutel, 2019; van Heijst & Geurts, 2015). It has been suggested that age at diagnosis is more critical to HRQOL compared to chronological age, with a later diagnosis being associated with reduced HRQOL and more negative outcomes possibly due to, for example, delayed therapeutic effects or delayed access to specific healthcare/ support services (Atherton et al., 2022; cf. Mason et al., 2018). Conversely, evidence on the predictive value of autism severity on HRQOL has also been mixed or weak (Kim & Bottema-Beutel, 2019; Leader et al., 2021; Renty & Roeyers, 2006; van Heijst & Geurts, 2015). Oakley and colleagues (2021) suggested that poorer HRQOL is not linked to autism per se but rather comorbidity burden (i.e., rates of depression). This is in accordance with the clinical observation that autistic adults often seek treatment not due to their “autism,” but because of somatic or mental health issues they experience. In fact, mental comorbidity, in particular, has now almost consistently been found as a significant negative predictor of HRQOL in autistic adults (Deserno et al., 2019; Knüppel et al., 2018; Mason, Mackintosh, et al., 2019; Park et al., 2019).

Despite increased comorbidity burden (Croen et al., 2015; Kohane et al., 2012; Lai et al., 2019; Vohra et al., 2017; Williams & Gotham, 2022) and the predictive value of comorbidity for HRQOL, numerous barriers to healthcare for autistic adults have been claimed, which hinder effective care for autistic adults. These exist at individual (e.g., communicative impairments in making appointments or describing symptoms), professional (e.g., lack of knowledge or reluctance to treat autism), and system-related levels (e.g., lack of services or inadequate services, limited access to existing services; Brede et al., 2022; David et al., 2022; Dern & Sappok, 2016; Duckert et al., 2023; Mason, Ingham, et al., 2019). Vogan and colleagues (2017) revealed that autistic adults with increased health concerns often show high general service use and use of emergency or inpatient care while reporting increased barriers to healthcare and lower satisfaction with services (also Gilmore et al., 2022). This suggests a healthcare system that does not meet the needs of autistic adults. In fact, barriers to accessing primary care in autism were significantly associated with not attending specialist referral when needed, requiring more intense treatment (e.g., surgery), or with serious health conditions due to delayed utilization of services (Doherty et al., 2022). Similarly, Brede and colleagues (2022) suggested that regular mental healthcare services do not adequately support or may even harm autistic adults.

Thus, there is reason to believe that barriers to healthcare have a direct impact on the subjective well-being of autistic adults. Unfortunately, statistical evidence on the direct relationship between barriers to healthcare and mental or physical HRQOL in autistic adults is lacking. A few studies now suggested a positive correlation between received family or social and other support—which often compensates for healthcare disparities—and HRQOL in autistic adults (Bishop-Fitzpatrick & Rubenstein, 2019; Charlton et al., 2023; Kamio et al., 2013; Khanna et al., 2014; Leader et al., 2021; Mason et al., 2018; Renty & Roeyers, 2006). Yet, there is only little evidence on the relationship between HRQOL and utilization of other specific healthcare services in autism (i.e., outpatient, inpatient, and emergency visits).

Thus, the present study aimed to (1) investigate the current mental and physical HRQOL of autistic adults in Germany and (2) analyze a comprehensive set of previously studied and novel predictors of both HRQOL dimensions including barriers to healthcare using multiple linear regression analyses. To our knowledge, this is the first study to investigate the statistical relationship between experienced barriers to healthcare and individual levels of mental or physical HRQOL. Identifying the most relevant predictors of HRQOL in autistic adults including potential healthcare barriers will not only help to identify vulnerabilities in autistic individuals but also vulnerabilities within the current healthcare system in order to inform healthcare providers and policymakers to improve healthcare and outcomes for autistic adults.

## Methods

### Participants

Participants were recruited across Germany using purposive, quota, and snowball sampling methods through the study’s collaborative network, publicly available contacts from autism-related and healthcare associations, local and countrywide outpatient clinics, local social media, and personal contacts. Participants were included in the online survey if they (1) were adults, (2) reported a professionally confirmed diagnosis of ASD with (3) no co-occurring intellectual disability (IQ > 70) as well as (4) sufficient language skills for the survey completion. Asking participants about a formerly confirmed clinical diagnosis of ASD, the diagnostic category, and diagnostic age has been shown to be highly predictive of the actual existence of the diagnosis (Baron-Cohen et al., 2015; Daniels et al., 2012). Participation was voluntary and anonymous. Informed consent was obtained prior to participation. No incentives were given for survey participation.

### Data collection and measures

The study was conducted in accordance with the WMA Declaration of Helsinki and was approved by the Local Psychological Ethics Committee of the Center for Psychosocial Medicine of the University Medical Center Hamburg-Eppendorf (ref. LPEK-0227). Data were collected, processed, and stored in accordance with the EU General Data Protection Regulation. Data were collected as part of a larger nationwide online survey (David et al., 2022), programmed in LimeSurvey GmbH (n.d.). Demographic, clinical, and utilization-related data were assessed as potential predictors of HRQOL (Table 1). In addition, participants completed the Short-Form Health Survey as operationalization of physical and mental HRQOL (SF-8; Beierlein et al., 2012; Ware et al., 2001) and the Barriers to Healthcare Checklist–Short Form (BHC-SF; Raymaker et al., 2017).

**Table 1.** Predictor variables and sample description.

	M (SD)	n (%)
*Demographic*		
Age (years)	38.9 (11.5)	
Gender		*Female*: 172 (55.3%) *Male*: 113 (36.3%) *Diverse*: 26 (8.4%)
Relationship status		*In a relationship (married/in a relationship)*: 79 (25.4%) *No relationship (single/divorced/ widowed)*: 232 (74.6%)
Education		*High school or equivalent (e.g., technical diploma)*: 218 (70.1%) *Lower or no degree (*secondary school/elementary school/special school/no degree*)*: 93 (29.9%)
Employment status		*Employed (employed full-time/part-time/in training)*: 138 (44.4%) *Not employed (unemployed/marginal employment/not applicable)*: 173 (55.6%)
*Clinical*		
Age at autism diagnosis (years)	32.5 (13.0)	
Required support due to social difficulties (DSM-5 interaction)	3.5 (1.1)	*Note*: rated on a 5-point Likert scale (1 = none, 2 = little, 3 = moderate, 4 = quite, 5 = very)
Required support due to restrictive behavior and interests (DSM-5 behavior)	3.3 (1.1)	*Note*: rated on a 5-point Likert scale (1 = none, 2 = little, 3 = moderate, 4 = quite, 5 = very)
Number of somatic comorbidities (last 6 months)	1.4 (1.1)	
Number of mental comorbidities (last 6 months)	1.5 (1.4)	
*Healthcare-related*		
Outpatient treatment (number of visits last 6 months)	21.0 (26.5)	
Inpatient treatment (number of days last 6 months)	4.1 (15.5)	
Emergency treatment (number of contacts last 6 months)	0.3 (0.9)	
Informal support (number of days last 6 months)	30.0 (54.2)	
Professional/ formal support (number of contacts last 6 months)	9.0 (25.2)	
*Barriers to Healthcare*		
BHC-SF sum score	8.1 (4.1)	

M: mean, SD: standard deviation. DSM-5: Diagnostic and Statistical Manual of Mental Disorders, 5th edition; BHC-SF: Barriers to Healthcare Checklist–Short Form.

#### HRQOL

HRQOL was assessed using the German version of the SF-8, for which German normative data were available (*n* = 2552; Beierlein et al., 2012). The SF-8 provides results equivalent to the SF-36. It contains eight items corresponding to eight subscales (Physical Functioning, Role Physical, Bodily Pain, General Health, Vitality, Social Functioning, Role Emotional, and Mental Health) rated on a 5-/6-point Likert-type scale. Rating subscales were used to calculate two summary scores indicative of mental and physical HRQOL (mental component summary score, MCS; physical component summary score, PCS). The MCS and PCS were then calculated as the weighted sum of the subscale scores. By using a norm-based scoring method, scores can be presented as standardized *T*-scores (with M = 50, SD = 10). The SF-8 has good to adequate psychometric properties in terms of overall reliability and validity (Beierlein et al., 2012; Yiengprugsawan et al., 2014). There is also evidence of psychometrically sound applications of the SF-8, SF-12, and SF-36 in autistic adults (Hayakawa et al., 2015; Khanna et al., 2015; van Heijst & Geurts, 2015).

#### Demographic variables

Participants provided information about age (years), gender (female, male, and diverse), relationship status (single, married/ in a relationship, divorced, widowed), highest educational degree/ attainment (high school diploma, secondary school, elementary school, special school, no degree) and employment status (full-time, part-time, student, unemployed, and other).

#### Clinical variables

Clinical variables included type of confirmed ASD diagnosis (autistic disorder, atypical autism, Asperger syndrome, other), diagnostic age (years), and presence of somatic and/or mental comorbidities (summed number). Self-rated autism symptom severity was assessed with two items analogous to the “Diagnostic and Statistical Manual of Mental Disorders” (4th ed.; DSM-5; American Psychiatric Association, 2013). Participants rated the following two items: “How much support do you need because of difficulties with interpersonal communication and social interactions?” (DSM-5 interaction) and “How much support do you need because you persist in behavioral habits, routines, or interests that are important to you (e.g., difficulties in self-organization or dealing with change)?” (DSM-5 behavior). Responses were given on a 5-point Likert-type scale (“none (1)—little (2)—moderate (3)—quite (4)—very (5)”).

#### Healthcare-related variables

Utilization history of professional healthcare, professional/ formal support services and informal support for the last 6 months were collected using modified versions of the German Questionnaire for the Assessment of Health Services in Old Age (FIMA; Seidl et al., 2015) and the Questionnaire on the Utilization of Medical and Nonmedical Care Services in Mental Disorders (FIMPsy; Grupp et al., 2018). These included outpatient treatment/care (summed visits), inpatient care (summed days), and emergency care (summed contacts). Informal support was assessed as the summed number of days that family members or friends provided help. Formal support included household help, social worker, nursing service/home care or legal custodians (summed number of contacts).

#### Barriers to healthcare

We used a translated version of the BHC-SF (Raymaker et al., 2017; Peth et al., submitted). The checklist was developed specifically for autistic people to assess barriers they often experience in clinical settings. It shows good content and construct validity (Raymaker et al., 2017). Barriers on the checklist relate to emotional difficulties, executive and sensory dysfunction, problems with healthcare navigation, patient-provider communication, provider attitudes, socioeconomic and social support limitations, as well as environmental factors (e.g., “I don’t understand the healthcare system” or “I find it hard to handle the waiting room”). The Short Form includes 17 items rated “yes” (=1) or “no” (=0), which are summed to a total score (minimum score: 0 = no barriers at all, maximum score: 17 = barriers in every aspect asked about).

### Data analysis and selection of predictors

Data were analyzed using Microsoft Excel and IBM SPSS Statistics (Version 27). Missing values in predictors were imputed using the Expectation-Maximization method implemented in SPSS. For somatic comorbidities 6.4%, for mental comorbidities 5.5%, for inpatient care 0.3%, for informal support 4.8%, and for formal support 4.8% of data were missing and replaced.

We performed three kinds of analyses. First, two-tailed independent *t*-tests were performed (unequal variances assumed, *p* < 0.05 considered significant) to determine whether the mean HRQOL for the MCS (i.e., mental HRQOL) and PCS (i.e., physical HRQOL) of the SF-8 in the autism group differed from the German normative sample (Beierlein et al., 2012). Second, bivariate correlational analyses (Pearson correlations between metric variables, point-biserial correlations between categorical and metric variables, Phi coefficients for correlations between categorical variables) were performed in order to (1) examine relationships between HRQOL and demographic, clinical, and healthcare-related variables and (2) to detect multicollinearity between predictors (as indicated by high correlations between two variables). Multicollinearity between predictors in our regression model was further assessed using the variance inflation factor (VIF). If two variables were highly correlated and had a critical VIF, as indicated by a VIF value > 5 (Menard, 2001), they were excluded from the multiple regression model. Interpretation of effect sizes/correlations was based on the conventions of Cohen (1988) (small/weak: *d* = 0.2/*r* = 0.10, medium/moderate: *d* = 0.5/*r* = 0.30, large/high: *d* = 0.8/*r* = 0.50).

Third, multiple regression analyses with either the MCS or PCS as dependent variable were conducted in order to identify significant predictors of mental and physical HRQOL that significantly explained variance in each of the SF-8 domains. The standardized beta coefficients (i.e. absolute value) were inspected in order to evaluate the strength of each predictor for explaining variance in the dependent variable. Categorical variables with more than two categories were recoded into dichotomous format, that is, gender (female = 1, male/diverse = 0), education (high school = 1, lower education/other = 0), marital status (1 = married/in a relationship, no relationship/other = 0), and employment status (1 = employed or in training, unemployed/other = 0). Predictors were entered into regression analyses in one block. Prior to multiple linear regression analyses, a power analysis was conducted online (using an online tool by Hemmerich, 2019) in order to determine the necessary sample size for our regression model to become significant, given the number of predictor variables planned. Previous evidence from regression analyses on HRQOL showed *R*²s between 0.03 and 0.60 (Lawson et al., 2020; Mason et al., 2018; Renty & Roeyers, 2006), which increased from small to large effect sizes when including disability- or support-related in addition to demographic predictors into the model. A conservatively calculated power analysis indicated that with an estimated *R*² = .08 (between small and medium effect; Cohen, 1988), a statistical power of 0.80, and a significance level of *α* = 0.05, a sample size of *n* = 236 would be sufficient with 16 predictors.

## Community involvement

Our neurodivergent research team engaged peer researchers as well as clinical psychologists specialized in ASD diagnosis and treatment. The online survey was designed in a participatory manner in consultation of autistic adults. Members of the autism community piloted and critically reviewed earlier versions of the survey. Research was generally conducted in close collaboration with relevant stakeholders.

## Results

### Sample description

Data from 311 autistic participants were included in this study. Of all participants, 269 (86.5%) reported a diagnosis of “Asperger syndrome,” 20 (6.4%) “atypical autism,” 11 (3.5%) “autistic disorder,” and 11 (3.5%) “other” (for further sample description, see Table 1).

### Mental and physical HRQOL compared to the German normative sample

**Figure 1.**
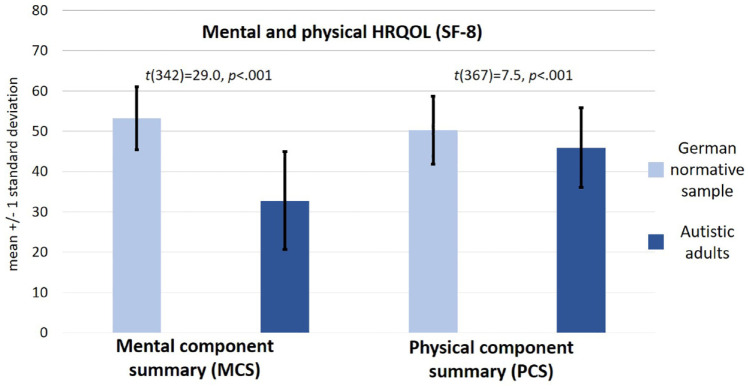
Mean scores of the mental and physical components of the SF-8 in our sample compared to the German normative sample (Beierlein et al., 2012). Legend: MCS as indicator of mental HRQOL, PCS as indicator of physical HRQOL. MCS and PCS represent standardized norm-based scores. Autistic adults (*n* = 311) in Germany showed significantly reduced HRQOL on both components of the SF-8 compared to the German normative sample (*n* = 2552; Beierlein et al., 2012). In particular, mental HRQOL was considerably below average in autistic participants.

Participants reported reduced mental HRQOL (M = 32.8, SD = 12.2) compared to the German normative sample (M = 53.3, SD = 7.8; Beierlein et al., 2012). As these mean scores represent normalized *T*-scores, this result also indicates that mental HRQOL in autistic adults was below average (more than 1.5 standard deviations below *T* = 50). This group difference was significant and corresponded to a large effect size (*t*(342) = 29.0, *p* < 0.001; Cohen’s *d* = 2.0; Figure 1).

Physical HRQOL was also reduced (M = 45.9, SD = 9.9, range = 20.5–65.2) compared to the German normative sample (M = 50.3, SD = 8.4; Beierlein et al., 2012). This group difference was also significant and corresponded to an almost moderate effect size (*t*(367) = 7.5, *p* < 0.001; Cohen’s *d* = 0.48; Figure 1).

### Correlation analyses

The full correlation matrix is shown in Table 2. *Mental HRQOL* showed moderate negative correlations with mental comorbidities (*r* = −0.404, *p* < 0.001) and the BHC-SF sum score (*r* = −0.416, *p* < 0.001). That is, mental HRQOL decreased with increasing number of mental comorbidities and the amount of self-reported barriers to healthcare. Similarly, *physical HRQOL* showed moderate negative correlations with somatic comorbidities (*r* = −0.462, *p* < 0.001) and the BHC-SF sum score (*r* = −0.420, *p* < 0.001). All other correlations between HRQOL components and the other variables were small or non-significant (Table 2).

**Table 2.** Correlation analyses.

	1.	2.	3.	4.	5.	6.	7.	8.	9.	10.	11.	12.	13.	14.	15.	16.	17.	18.
1. Mental HRQOL (MCS)	-	0.068	0.008	−0.040	0.016	−0.052	**0.180** ^**^	−0.066	**−0.142** ^*^	**−0.207** ^***^	**−0.135** ^*^	**−0.404** ^***^	**−0.132** ^*^	**−0.213** ^***^	**−0.126** ^*^	−0.069	**−0.150** ^**^	**−0.416** ^***^
2. Physical HRQOL (PCS)		-	**−0.221** ^***^	−0.111	−0.087	0.100	**0.126** ^*^	**−0.231** ^***^	**−0.149** ^**^	**−0.203** ^***^	**−0.462** ^***^	**−0.263** ^***^	**−0.193** ^**^	**−0.128** ^*^	−0.089	**−0.231** ^***^	**−0.119** ^*^	**−0.420** ^***^
3. Age			-	0.039	**0.207** ^**^	0.085	**0.138** ^*^	**0.830** ^***^	0.066	0.003	**0.194** ^**^	−0.067	0.052	−0.040	**−0.117** ^*^	0.013	−0.102	0.078
4. Gender				-	0.064	0.105	−0.017	0.099	−0.097	−0.086	0.102	−0.043	0.026	−0.054	−0.095	0.039	0.101	0.111
5. Relationship status					-	0.058	0.088	**0.274** ^***^	−0.079	−0.011	0.031	−0.059	−0.089	−0.066	−0.005	**0.180** ^**^	−0.106	−0.008
6. Education						-	0.103	0.091	−0.041	**−0.115** ^*^	0.046	0.025	0.038	0.057	−0.016	−0.094	0.000	−0.013
7. Employment status							-	**0.150** ^**^	−0.031	−0.191**	−0.097	**−0.137** ^*^	−0.111	−0.027	−0.053	−0.021	**−0.111** ^*^	**−0.146** ^**^
8. Diagn. age								-	0.057	0.009	**0.177** ^**^	0.005	0.065	−0.022	−0.104	−0.092	−0.025	−0.074
9. *DSM*-5 interaction									-	**0.438** ^***^	−0.031	**0.155** ^**^	**0.200** ^***^	0.105	−0.009	0.022	0.087	**0.168** ^**^
10. *DSM*-5 behavior										-	0.081	**0.261** ^***^	**0.220** ^***^	0.023	0.076	**0.122** ^*^	**0.193** ^**^	**0.296** ^***^
11. Somatic comorbidities											-	**0.293** ^***^	**0.227** ^***^	**0.153** ^**^	0.101	−0.051	**0.179** ^**^	**0.218** ^***^
12. Mental comorbidities												-	**0.185** ^**^	**0.154** ^**^	**0.189** ^**^	**0.136** ^*^	**0.126** ^*^	**0.344** ^***^
13. Outpatient treatment													-	0.068	**0.136** ^*^	0.057	**0.211** ^***^	**0.160** ^**^
14. Inpatient treatment														-	**0.154** ^**^	−0.046	−0.019	0.044
15. Emergency treatment															-	0.069	0.044	**0.112** ^*^
16. Informal support																-	0.067	**0.206** ^***^
17. Formal support																	-	**0.211** ^***^
18. BHC-SF																		-

SF-8: Short-Form Health Survey; MCS: mental component summary score of the SF-8; PCS: physical component summary score of the SF-8; DSM-5: Diagnostic and Statistical Manual of Mental Disorders, 5^th^ edition; BHC-SF: Barriers to Healthcare Checklist–Short Form.

* *p* < 0.05; ^**^p < 0.01; ^***^p < 0.001.

*Analyses between predictor variables*, and with respect to multicollinearity, revealed that age was significantly and highly correlated with age at diagnosis (*r* = 0.830, *p* < 0.001). Variables were kept for the multiple regression analysis after inspection of the VIF in the regression model (VIF < 5; Table 3). Furthermore, moderate positive correlations were detected between both DSM-5 scales (*r* = 0.438, *p* < 0.001) and between the number of mental comorbidities and the BHC-SF sum score (*r* = 0.344, *p* < 0.001). All other correlations between predictor variables were small or non-significant (Table 2).

**Table 3.** Multiple regression analyses (*N* = 311).

	*Mental HRQOL (MCS)*					*Physical HRQOL (PCS)*					
	*b*	*SE*	*95% CI*	*ß*	*p*	*b*	*SE*	*95% CI*	*ß*	*p*	*VIF*
*Demographic predictors*											
Age	0.14	0.09	[−0.05, 0.33]	0.13	0.145	−0.04	0.07	[−0.19, 0.10]	−0.05	0.552	3.42
Gender	−0.44	1.25	[−2.89, 2.01]	−0.02	0.724	−0.91	0.95	[−2.77, 0.96]	−0.05	0.339	1.10
Relationship status	0.51	1.48	[−2.40, 3.43]	0.02	0.728	−1.35	1.13	[−3.56, 0.87]	−0.06	0.232	1.19
Education	−1.56	1.33	[−4.17, 1.05]	−0.06	0.240	**2.70**	**1.01**	**[0.71, 4.68]**	**0.13**	**0.008**	1.06
Employment status	**2.73**	**1.25**	**[0.27, 5.19]**	**0.11**	**0.030**	0.95	0.95	[−0.92, 2.82]	0.05	0.318	1.11
*Clinical predictors*											
Diagnostic age	**−0.17**	**0.08**	**[−0.34, 0.00]**	**−0.18**	**0.044**	−0.09	0.06	[−0.21, 0.04]	−0.12	0.176	3.49
*DSM*-5 interaction	−0.26	0.62	[−1.49, 0.96]	−0.02	0.673	−0.75	0.47	[−1.69, 0.18]	−0.08	0.113	1.33
*DSM*-5 behavior	−0.30	0.62	[−1.52, 0.93]	−0.03	0.636	−0.03	0.47	[−0.96, 0.91]	0.00	0.957	1.45
Somatic comorbidities	0.45	0.60	[−0.74, 1.63]	0.04	0.459	**−2.95**	**0.46**	**[−3.85, −2.05]**	**−0.33**	**<0.001**	1.30
Mental comorbidities	**−2.06**	**0.48**	**[−3.00, −1.12]**	**−0.24**	**<0.001**	−0.22	0.36	[−0.93, 0.50]	−0.03	0.552	1.36
*Healthcare-related predictors*											
Outpatient treatment	0.00	0.02	[−0.05, 0.04]	−0.01	0.897	−0.01	0.02	[−0.04, 0.03]	−0.02	0.749	1.19
Inpatient treatment	**−0.12**	**0.04**	**[−0.20, −0.04]**	**−0.16**	**0.002**	−0.04	0.03	[−0.10, 0.01]	−0.07	0.142	1.09
Emergency treatment	−0.31	0.72	[−1.73, 1.11]	−0.02	0.666	−0.20	0.55	[−1.28, 0.87]	−0.02	0.712	1.10
Informal support	−0.02	0.01	[−0.04, 0.01]	−0.07	0.199	−0.01	0.01	[−0.03, 0.01]	−0.06	0.205	1.16
Formal support	0.02	0.03	[−0.03, 0.07]	0.04	0.479	**−0.04**	**0.02**	**[−0.08, 0.00]**	**−0.10**	**0.045**	1.15
*Barriers to healthcare*											
BHC-SF	**−0.87**	**0.17**	**[−1.19, −0.54]**	**−0.29**	**<0.001**	**−0.62**	**0.13**	**[−0.86, −0.37]**	**−0.25**	**<0.001**	1.32

MCS: mental component summary score of the SF-8; PCS: physical component summary score of the SF-8; b: unstandardized coefficient, SE: standard error, CI: confidence interval for b, ß: standardized coefficient (beta), VIF: variance inflation factor. DSM-5: Diagnostic and Statistical Manual of Mental Disorders, 5^th^ edition; BHC-SF: Barriers to Healthcare Checklist–Short Form.

## Multiple linear regression analyses

### Predictors of mental HRQOL

The overall regression model with all 16 predictors was statistically significant (*F*(16, 294) = 8.08, *p* < 0.001) and explained 26.8% of the variance in the mental HRQOL component (*R*^2^ = 0.305; adj. *R*^2^ = 0.268), indicating a high goodness of fit (Cohen, 1988). Number of Barriers to healthcare as assessed by the BHC-SF, were awere significant and strongest negative predictors of mental HRQOL (*ß* = −0.29, *p* < 0.001; Table 3), followed by the number of mental comorbidities (*ß* = −0.24, *p* < 0.001), age at diagnosis (*ß* = −0.18, *p* = 0.044) and days of inpatient treatment (*ß* = −0.16, *p* = 0.002). Fourth, employment status was a significant positive predictor of mental HRQOL (i.e., being employed was associated with higher mental HRQOL; *ß* = 0.11, *p* = 0.03).

### Predictors of physical HRQOL

The overall regression model with all 16 predictors was statistically significant (*F*(16, 294) = 11.85, *p* < 0.001) and explained 35.9% of the variance in the physical HRQOL component (*R*^2^ = 0.392; adj. *R*^2^ = 0.359), indicating a high goodness of fit (Cohen, 1988). The number of somatic comorbidities was a significant and strongest negative predictor of physical HRQOL (*ß* = −0.33, *p* < 0.001; Table 3), followed by the number of self-reported barriers to healthcare (*ß* = −0.25, *p* < 0.001) and the amount of professional/ formal support (*ß* = −0.10, *p* = 0.045). Educational attainment was a significant positive predictor of physical HRQOL (i.e., having a high school diploma or equivalent was associated with higher HRQOL; *ß* = 0.13, *p* = 0.008).

## Discussion

The present study provides a current picture of mental and physical HRQOL in autistic adults without co-occurring intellectual disability across Germany. Autistic adults reported significantly reduced mental (which was far below average) and physical HRQOL. Several independent variables had a significant effect for mental or physical HRQOL such as mental and somatic comorbidity rates. Barriers to healthcare emerged as the independent and only predictor that significantly explained variation in both HRQOL domains: The more barriers autistic adults experienced, the lower their mental and physical HRQOL. Particularly for mental HRQOL, healthcare barriers emerged as the strongest predictor. This is the first study to shed light on the impact of healthcare barriers on HRQOL in autistic adults.

### Reduced HRQOL in autistic adults in Germany

Here, autistic adults without intellectual disability in Germany reported significantly poorer physical and mental HRQOL compared to a German normative sample. Particularly, mental HRQOL was far below average (Figure 1). Our finding is in accordance with the notion of increased psychiatric comorbidity burden in autistic adults (Croen et al., 2015; Kohane et al., 2012; Lai et al., 2019; Vohra et al., 2017) and with previous evidence on particularly compromised (mental) HRQOL in autistic adults using other versions of the SF (Braden et al., 2022; Helles et al., 2017; Khanna et al., 2014). For example, Khanna and colleagues (2014) reported that autistic adults in the United States showed significantly lower mental HRQOL on the SF-12 compared to the general U.S. population. This effect was found across age and gender, whereas physical HRQOL was significantly reduced as a function of age. Unfortunately, we also did not include a matched comparison group, but compared HRQOL in our sample of autistic adults with German normative data. Our finding is in line with Braden and colleagues (2022), who—using the SF-36—found HRQOL to be significantly reduced in both autistic men and women compared to a matched sample of neurotypical participants.

Differential modulations of mental versus physical HRQOL may suggest different factors associated with either mental or physical well-being in autism, also implying possibly different target groups (e.g., older vs. younger individuals and women vs. men) or actions when aiming at improving mental or physical HRQOL in autistic adults. Multiple linear regression analysis helps to identify healthcare priorities in autistic adults (“understanding what works for whom and when, and what are some of the predictable needs and variations that need to be considered to support autistic individuals,” Lord et al., 2022).

### Unique predictors of mental or physical HRQOL in autistic adults: demographic, clinical, and healthcare-related variables

Here multiple regression analyses revealed non-overlapping sets of predictors of either mental or physical HRQOL in our autistic participants. Autistic adults who were particularly vulnerable to lower mental HRQOL more often reported unemployment, later diagnosis of ASD, more mental health comorbidities, and greater use of inpatient treatment. Those who were particularly vulnerable to reduced physical HRQOL reported lower educational attainment, more somatic comorbidities, and increased use of professional support services.

*With respect to demographic predictors*, employment status has previously been associated with HRQOL in autistic adults (e.g., Khanna et al., 2014; Knüppel et al., 2018; Mason et al., 2018). Here, being employed positively predicted mental HRQOL. Mental illness, particularly depression, has been related to unemployment in autistic adults (Park et al., 2019). This notion was supported by our data, which showed weak negative correlations between being employed and the number of mental comorbidities as well as autism-related behavioral difficulties (Table 2). Discrepancies with other studies, which have not linked employment status to (mental) HRQOL (Kamio et al., 2013; for an overview, van Heijst & Geurts, 2015) might be explained by differences in sample constitutions. Unemployment rates were increased in our sample (Table 1). For physical HRQOL, higher educational attainment emerged as a significant positive demographic predictor in autistic adults (see also Mason et al., 2018). Educational attainment has previously been associated with healthcare utilization and self-care behavior in other physical health conditions (e.g., Alguwaihes & Shah, 2009). Educational attainment in our sample was high (Table 1), potentially compensating and weakening the effect of reduced physical HRQOL in contrast to the mental component (Figure 1). Our bivariate data analyses further showed weak negative correlations between educational attainment and autism-related behavioral difficulties. These might also affect individual health management (incl. the use of preventive services) or imply increased barriers to accessing healthcare, making external informal or formal support for healthcare necessary (Table 2).

*With respect to clinical predictors*, diagnostic age negatively predicted mental HRQOL, that is, participants who received an ASD diagnosis later in life reported lower mental HRQOL (Atherton et al., 2022). Although our sample also included participants who were diagnosed at <18 years of age (15% of participants), on average, participants were diagnosed in their thirties (Table 1). Early diagnosis may be an indicator of greater severity of autism, but it also represents a door opener to specific interventions and support services critically important to promoting health and other outcomes (Burke & Taylor, 2023; Skaletski et al., 2021). In fact, late diagnosis of ASD has been associated with increased or more severe mental health conditions, which—if left untreated—lead to alarmingly poor outcomes (Croen et al., 2015; Lai et al., 2019; Maddox & Gaus, 2019; Mandy et al., 2022). It is therefore not surprising that, here, the number of self-reported mental comorbidities (in line with Helles et al., 2017; Lawson et al., 2020; Mason et al., 2018) negatively predicted mental HRQOL in our participants (while the number of somatic comorbidities negatively predicted physical HRQOL; compare Charlton et al., 2023).

*With respect to healthcare-related predictors*, only the duration of inpatient treatment emerged as a significant, negative predictor of mental HRQOL, while contacts to professional support services emerged as a significant, negative predictor of physical HRQOL. Although inpatient care has previously also been associated with improved outcomes for autistic individuals (Melvin et al., 2022), psychiatric inpatient admission has typically been linked to increased autism severity and mental health comorbidity burden (Righi et al., 2018). Use of inpatient treatment, thus, might indicate increased mental healthcare needs in autism. Indeed, there was a weak positive correlation between duration of inpatient care and comorbidity rates (Table 2), supporting the notion that inpatient treatment reflects more severe or complex mental health problems, and is therefore associated with poorer mental HRQOL. Conversely, adverse health effects of mental healthcare services themselves in autistic adults have been discussed (Brede et al., 2022) as well as the possibility that utilization of inpatient treatment and associated severe health problems may arise from barriers at the primary care level (Doherty et al., 2022). Unfortunately, the cross-sectional nature of our data does not allow us to pin down the exact nature of the relationship between inpatient treatment and mental HRQOL, that is, as mediated by psychiatric comorbidities or ineffective healthcare provision in autistic adults.

For physical HRQOL, formal support contacts emerged as a specific negative predictor. These included nursing service or home care, often granted when a person needs care due to physical illness and being dependent on the help of others. As such, the use of formal support services might also be specifically indicative of more severe physical health problems, hence showing an association with worse physical HRQOL (see Table 2 for positive correlations between formal support utilization and autism severity, comorbidities, and outpatient treatment). On the other hand, increased support service utilization—while physical health issues persist or worsen—has also been interpreted as evidence for barriers to accessing more basic care services and for unmet healthcare needs in autism (e.g., Gilmore et al., 2022; Renty & Roeyers, 2006).

Our findings suggest that facilitation of early diagnosis, supported employment, and effective, early treatment should be key priorities in order to improve HRQOL in autistic adults—particularly mental HRQOL—which was seriously affected in our sample. These may include education of practitioners and employers for adult autism, increased availability of autism-specific diagnostic and therapeutic structures for adults, and autism-specific supported employment (see also Duckert et al., 2023; Mason, Ingham, et al., 2019).

Barriers to healthcare predict both lower mental and physical HRQOL in autistic adults. Whereas different independent positive and negative predictors were associated with either mental or physical HRQOL in autistic adults, healthcare barriers negatively predicted both. Autistic adults in Germany experience clinically relevant barriers to healthcare. Especially for reduced mental HRQOL, barriers emerged as the strongest predictor (in line with Brede et al., 2022, who emphasized several adverse effects of current mental health services for autistic adults’ mental well-being). Standardized beta coefficients were inspected in order to compare the strength of the effect of each independent variable to the dependent variable, with higher absolute coefficients indicating stronger effects on HRQOL. In doing so, barriers emerged as the strongest independent predictor for mental HRQOL (after mental comorbidities) and the second-strongest predictor for physical HRQOL (for which physical comorbidities showed the strongest effect).

Self-reported barriers to healthcare were correlated with increased somatic and, especially, mental comorbidity, with autism severity, unemployment, increased outpatient and emergency treatment as well as informal and formal support. While the negative relationship between barriers to healthcare and present health problems can quite intuitively be understood, the positive relationship between such barriers and healthcare utilization seems less intuitive. Yet, the present findings are consistent with previous evidence on high general service use or emergency/ hospital treatment in autism and concurrent experience of healthcare barriers as well as lower service satisfaction in autism (e.g., Brede et al., 2022; Doherty et al., 2022; Gilmore et al., 2022; Renty & Roeyers, 2006; Vogan et al., 2017). Formal and, particularly, informal support (by family members) thereby often compensates for healthcare barriers in autism by aiding navigation within the healthcare system, facilitating access to healthcare or assisting patient-provider communication. Our findings underscore the notion of unmet healthcare needs in autism (Brede et al., 2022; Platos & Pisula, 2019), which is further underlined by the recent dramatic finding that autistic adults in the United Kingdom receive poorer quality healthcare than those without autism (Weir et al., 2022).

Barriers to healthcare here were measured using the BHC-SF (Raymaker et al., 2017). Compared to a previous study using the BHC-SF in the United States, our autistic participants on average reported increased barriers to healthcare (with a mean sum score on the BHC-SF that was almost double the mean score reported by Nicolaidis et al., 2016). This quantifies and corroborates previous qualitative findings from Germany and other countries (Calleja et al., 2022; Dern & Sappok, 2016; Duckert et al., 2023) but also points to particular shortcomings of the healthcare system for autistic adults in Germany (possibly similar to the United Kingdom as cited above; Weir et al., 2022). Public and clinical awareness of autism in adulthood in Germany has increased relatively recently. As such, we know little about prevalence rates or other epidemiological data on adult autism in Germany for the last 20 years (Roy & Strate, 2023). Unfortunately, from the present data, it remains unclear where the increased rates of self-reported barriers in our German sample arise from. The cross-sectional correlational findings do not allow inferences about the direction of relationships. As such, comorbidity rates, autism severity, or delayed diagnosis of autism could have contributed to increased access barriers or vice versa.

The present ratings on the BHC-SF (Raymaker et al., 2017) provide information about specific ways in which healthcare for autistic adults in Germany—but not only here—could be improved. Our findings suggest that reducing barriers to healthcare could lead to improved HRQOL and reduced adverse outcomes in autistic adults. There is also good reason to believe that reducing barriers might lead to more effective and less costly healthcare in autism (Doherty et al., 2022; Höfer et al., 2022).

### Limitations

There are several limitations to our study. First, the present cross-sectional observational study is limited in its ability to make causal inferences. That is, although here barriers to healthcare significantly predicted HRQOL in autistic adults, we cannot draw the conclusion that barriers *cause* lower HRQOL. Our results also do not address possible nested, mediated, or interaction effects between predictor variables (e.g., between barriers to healthcare and mental health conditions). As such, our findings may reflect different mechanisms— which are likely to be complex in autistic adults—associated with barriers to healthcare on the one hand and HRQOL on the other. Future research, possibly longitudinal or intervention studies, will be necessary to tease these apart to inform successful health care for autistic adults.

Second, we cannot rule out sampling bias as our data are based on self-report by autistic adults with online access and sufficient cognitive and language skills to complete the survey and reflect on their own health situation. By definition, HRQOL is a patient-reported outcome for which self-report is key. Our sample constitution and results are comparable to those of previously published HRQOL studies. Yet, our results do not represent the entire autism spectrum (e.g., early-diagnosed participants, autistic adults with intellectual disability). In Germany, but also internationally, there are specific services for autistic adults with but not without intellectual disability, who—being considered “high-functional” and with disabilities not being obvious—are often confronted with so-called invisible barriers in accessing or finding the much-needed healthcare or support services within regular healthcare structures (Beresford et al., 2020; Brede et al., 2022). Accordingly, there is now growing evidence that autistic adults without intellectual disabilities often show especially poor health or employment outcomes (Taylor & Seltzer, 2011). Unfortunately, our data did not allow comparisons between sub-groups of autistic adults with and without intellectual disability or other comparison groups (especially equally underserved patient populations with unmet healthcare needs).

## Conclusions and implications

Autistic adults in Germany experience reduced mental and physical HRQOL and significant barriers to accessing the healthcare they need. The present study is the first to demonstrate a direct link between the barriers to healthcare autistic adult's experience and their mental and physical HRQOL. Reducing barriers to healthcare along with effective treatment and prevention of mental and somatic illness and facilitation of employment and education were identified as the highest priorities for actions in order to improve the health status of autistic adults. In light of the UN Convention on the Rights of Persons with Disabilities (2006), which calls for equal rights for people with disabilities, including equal access to and quality of healthcare, the present findings have alarming and urgent implications for the healthcare system, service providers, health insurers, and policymakers, who must finally become aware of the barriers and needs autistic adults in seeking healthcare.
